# Considerations for multidisciplinary management of synchronous primary breast cancer and primary lung cancer – Analysis of thirty‐one patients

**DOI:** 10.1111/1759-7714.15284

**Published:** 2024-04-04

**Authors:** Xue Chen, Yi‐fan Fang, Wan‐Pu Yan

**Affiliations:** ^1^ Key Laboratory of Carcinogenesis and Translational Research (Ministry of Education), Breast Center Peking University Cancer Hospital & Institute Beijing China; ^2^ Department of Thoracic Surgery I, Key Laboratory of Carcinogenesis and Translational Research (Ministry of Education) Beijing Cancer Hospital, Peking University School of Oncology Beijing China

**Keywords:** breast cancer, lung cancer, primary lung cancer, synchronous primary breast cancer

## Abstract

**Background:**

The simultaneous (synchronous) presence of primary breast cancer and primary lung cancer diagnosed in a single individual is not an uncommon phenomenon. However, reference data for treatment strategy is scarce and “chaotic”. In the present study we discuss the management strategy for this group of patients.

**Methods:**

We retrospectively reviewed patients in the primary breast cancer database of the Breast Center and the primary lung cancer database of the Thoracic Surgery Department I of Peking University Cancer Hospital. Patients with synchronous primary breast cancer and primary lung cancer who underwent surgery between December 2010 and December 2023 were included in the study. The sequence of outpatient visits, recommendations of multidisciplinary teams, perioperative treatment, and surgical procedures were reviewed. Meanwhile, survival analysis based on propensity score matching with 1:1 ratio was performed between the 31 patients and those with lung cancer only during the same period.

**Results:**

A total of 31 patients with synchronous primary breast cancer and primary lung cancer were identified; all of the patients were women. The average age was 61 years. A total of 24 of the patients had visited the breast center first, and routine chest computed tomography (CT) showed evidence of primary lung cancer. The other seven patients had visited the thoracic surgery clinic first, and routine positron emission tomography (PET)‐CT revealed the coexistence of primary breast cancer. All the patients had multidisciplinary team consultations, after which 20 patients were recommended to have preoperative treatment for breast cancer, two patients were recommended to have preoperative treatment for lung cancer, and nine patients were recommended to undergo surgery directly. After surgery, 23 patients received postoperative adjuvant treatment for breast cancer, and no patients needed postoperative adjuvant treatment for lung cancer. Survival analysis showed that there was no significant difference between the 31 patients and those with lung cancer only.

**Conclusion:**

Routine chest CT is needed for breast cancer patients before surgery, and PET‐CT is required for the accurate staging of lung cancer patients. A multidisciplinary expert team should manage synchronous primary breast cancer and primary lung cancer. Emphasis should be placed on patients who need preoperative treatment before surgery. Particularly, for patients who need preoperative chemotherapy, a regimen should be chosen that balances the treatment of lung cancer and breast cancer.

## Graphical abstract

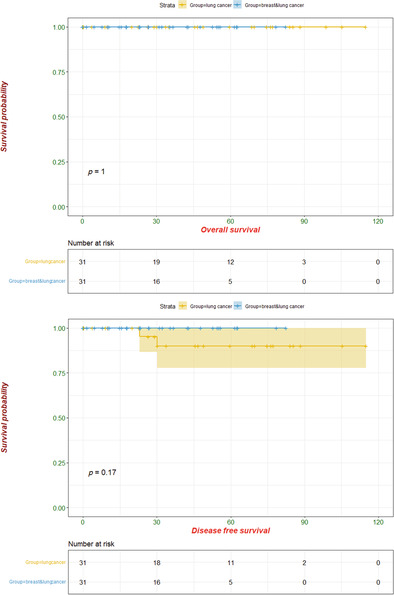

## INTRODUCTION

Among women, breast cancer has the highest incidence worldwide, 29.05/100 000; lung cancer has the highest mortality among all malignancies, 28.09/100 000.^1^ When breast cancer and lung cancer occur in the same patient, there might be some confusion about the choice of treatment strategy. Primary lung cancer has a high incidence, and the lung is also a frequent metastatic site of most solid cancers, including breast cancer. Therefore, for breast cancer patients, we should not ignore the possibility of primary lung cancer nor forget the basic fact that breast cancer is prone to pulmonary metastasis.^2^ Comprehensive treatment of breast cancer has achieved great success, which can be called the epitome of the progress in solid tumor treatment. Preoperative induction therapy is also greatly improving the long‐term survival of lung cancer patients. When primary breast cancer and primary lung cancer occur synchronously in the same patient, it is critical to comprehensively consider the treatment strategy based on TNM staging, molecular characteristics, precision diagnosis, and multidisciplinary discussion. However, limited relevant data exists in the literature, and the available treatment references are limited. Therefore, chaotic treatment approaches are inevitable. In the present study, we summarize the clinical experience in 31 synchronous primary breast cancer and primary lung cancer patients from our hospital.

## METHODS

The primary breast cancer database of our Breast Center was established in 1998, and the primary lung cancer database of Thoracic Surgery Department I was established in 2000. Both databases are prospective databases. For this study, patients who received treatment from December 2010 to December 2023 and patients with synchronous primary breast cancer and primary lung cancer who received surgical treatment were included. The performance status and clinicopathological characteristics of patients were recorded. The sequence of outpatient visits, recommendations of multidisciplinary teams, perioperative treatment, and surgical procedures were reviewed. Meanwhile, survival analysis based on propensity score matching with 1:1 ratio was performed between the 31 patients and those with lung cancer only during the same period.

1. Management of breast cancer in our hospital:^3^, ^4^, ^5^, ^6^
  - Qualitative diagnosis of breast cancer, including molecular classification testing related to pathology and treatment: hormone‐receptor positive (luminal A/B), Her2 status, triple‐negative.
  - Staging work‐up: chest/abdominal computed tomography (CT) and bone scan. Patients also had magnetic resonance imaging (MRI) to exclude multiple lesions.
  - For triple‐negative tumors with largest diameter >1 cm, chemotherapy was recommended before surgery. For Her‐2‐positive tumors with largest diameter >1 cm, chemotherapy + targeted therapy was recommended before surgery. For luminal A/B with positive lymph nodes or the largest diameter of primary tumor >5 cm, chemotherapy was recommended before surgery.
2. Management of lung cancer in our hospital:^7^, ^8^, ^9^, ^10^, ^11^
  - Except for stage IA peripheral lung cancer without enlarged hilar lymph node, preoperative biopsy pathological diagnosis and treatment‐related molecular screening were required for all suspected lung cancers.
  - Quantitative (TNM staging) diagnosis included chest contrast CT, bronchoscopy, brain MRI, liver and supraclavicular ultrasound, and, most importantly, routine positron emission tomography (PET)‐CT. For suspicious enlarged lymph nodes in the bilateral supraclavicular region, bilateral mediastinum or hilum, especially with the primary lesion located in the center, endobronchial ultrasound (EBUS) biopsy was required.
  - Stage I lung cancer patients underwent surgery directly. Principally, preoperative treatment based on chemotherapy was required for patients with stage II/IIIA lung cancer.

## RESULTS

A total of 31 patients with synchronous primary breast cancer and primary lung cancer were included in the study. All the patients were women, aged 52 to 78, with an average age of 61. A total of 24 patients visited the breast center first, and routine chest CT revealed the possibility of primary lung cancer. Seven patients visited thoracic surgery first, and routine PET‐CT revealed the possibility of primary breast cancer. All the patients had multidisciplinary team consultations, after which 20 patients were recommended to have preoperative treatment for breast cancer, including six triple‐negative breast cancer patients who received chemotherapy followed by surgery, four patients with Her‐2 overexpression who received preoperative chemotherapy + targeted therapy followed by surgery, 10 patients with luminal A/B breast cancer who received endocrine therapy during the treatment for lung cancer. Two patients were recommended to have preoperative treatment for lung cancer, and nine patients were recommended to undergo surgery directly. After surgery, 24 patients received postoperative adjuvant treatment for breast cancer, and no patients needed postoperative adjuvant treatment for lung cancer.

A total of 13 breast cancer patients had the lesions on the left side, and 18 were on the right side. There were 24 patients with positive hormone‐receptor (luminal A or luminal B), three with triple‐negative, and four with Her‐2 overexpression. For clinical stages, one patient had clinical TisN0M0, 13 patients had clinical T1N0M0, and 17 patients had clinical T2N0M0. For surgical procedures, 13 patients underwent breast conserving surgery, 15 underwent simple mastectomy surgery, and three underwent modified radical mastectomy. A total of 27 patients received postoperative adjuvant treatment, including 22 with endocrine therapy, four with targeted therapy, and one with chemotherapy.

For lung cancer patients, the histopathological subtypes of all the patients were adenocarcinoma. Five patients had lesions in the right upper lobe, eight were in the right middle lobe, five in the right lower lobe, nine in the left upper lobe and four in the left lower lobe. With regard to stages, two patients were TisN0, eight were T1aN0, one was T1aN0, one was T1bN0, five were T1bN0, and 14 were T1cN0. For surgical procedures, 19 patients underwent lobectomy, and 12 patients underwent sublobectomy. No patient received postoperative adjuvant treatment for lung cancer.

Survival analysis was based on propensity score matching (PSM) with 1:1 ratio between the 31 patients and those with lung cancer only during the same period.

A total of 31 patients with lung cancer only were included in the PSM, all of which were with adenocarcinoma. With regard to locations, seven were in the right upper lobe, five were in the right middle lobe, five were in the right lower lobe, eight were in the left upper lobe, and six were in the left lower lobe. For staging, three were TisN0, seven were T1aN0, 16 were T1bN0 and five were T2aN0. For surgical procedures, 18 were lobectomies and 13 were sublobectomies. No patient received postoperative adjuvant treatment.

**FIGURE 1:**
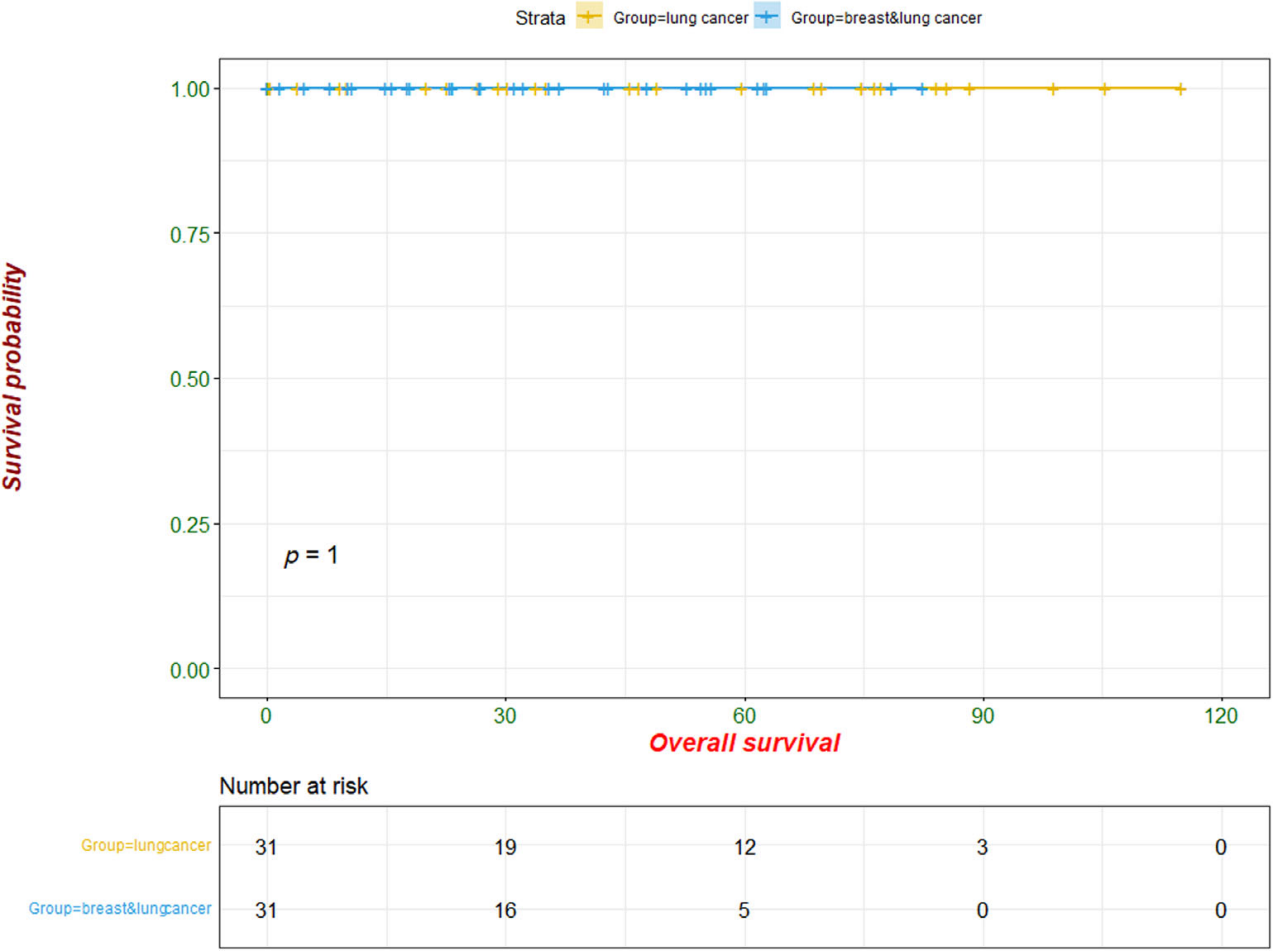
Overall survival analysis between the 31 simultaneous primary lung cancer/primary breast cancer patients and the matched 31 patients with primary lung cancer only.

**FIGURE 2:**
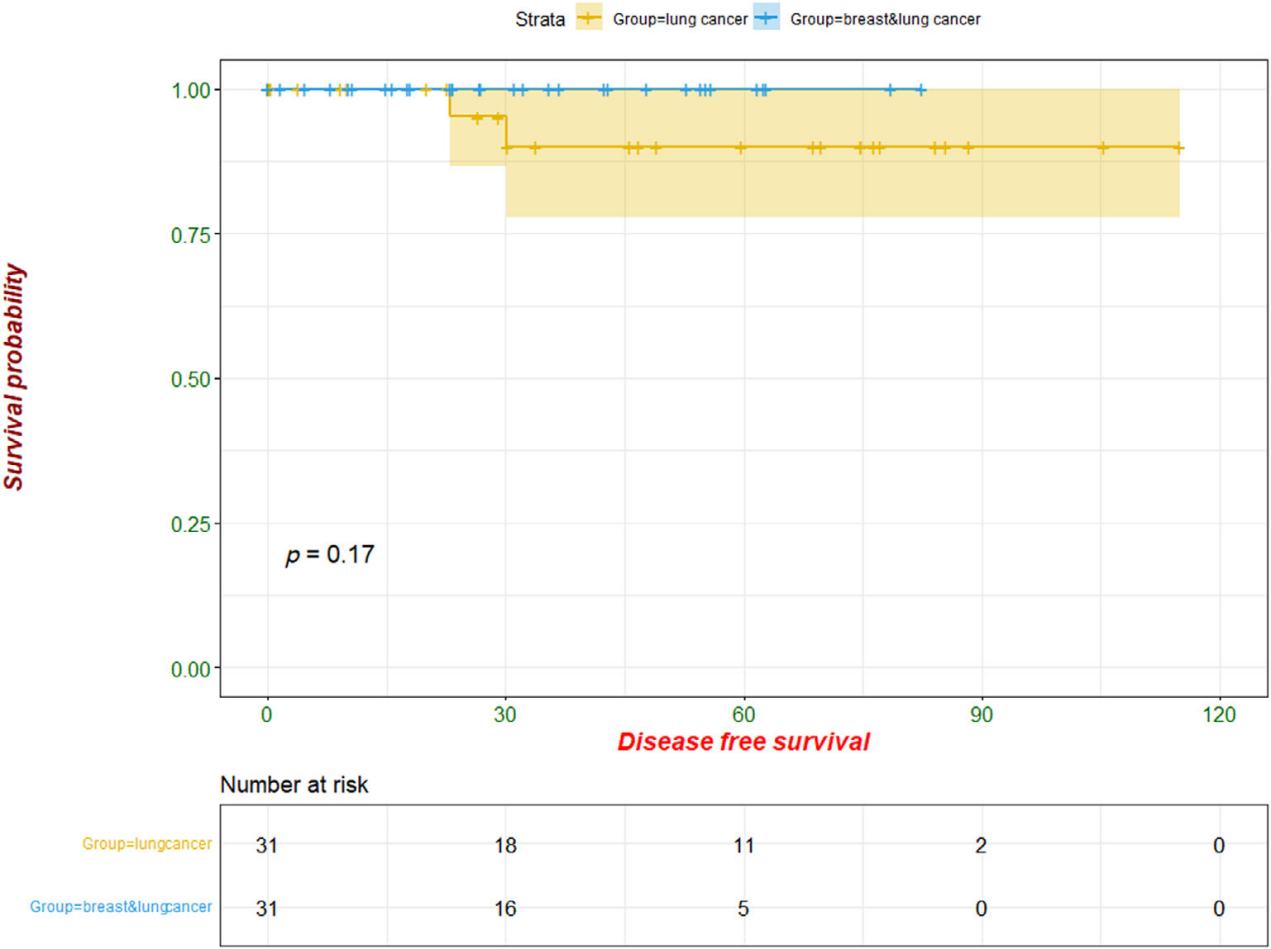
Disease‐free survival analysis between the 31 simultaneous primary lung cancer/primary breast cancer patients and the matched 31 patients with primary lung cancer only.

During follow‐up (to December 2023), none of the 31 patients had local recurrence or distant metastasis. PSM survival analysis showed that the DFS and OS were comparable between the 31 patients with simultaneous lung cancer and breast cancer, and those with lung cancer only (Figures 1 and 2).

## DISCUSSION

### In patients with malignancies, attention should be focused on systemic examination

Among women, breast cancer has the highest incidence (29.05/100 000 worldwide), and lung cancer has the highest mortality of all malignancies (28.09/100 000).^1^ The detection rate of lung adenocarcinoma presenting as ground‐glass opacity (GGO) components in imaging also appears to be increasing for nonsmoking women.^12^ In recent years, the incorporation of low‐dose CT for high‐risk populations marked huge progress in lung cancer screening. A previous study proved that low‐dose CT decreased the mortality of lung cancer by 20%.^13^ High risk populations include persons aged >50, smokers, patients with a family history of lung cancer and history of other malignancies. In this sense, breast cancer patients belong to a population that has a high risk for lung cancer. Therefore, as breast cancer surgeons, we should keep in mind that lung cancer has a high incidence, and the organ itself is also one of the most frequent sites for solid cancer metastasis, including breast cancer. For breast cancer patients, we should not ignore the possibility of frequently occurring primary lung cancer, nor forget the basic fact that breast cancer is prone to lung metastasis.^2^ In this study, most patients visited a breast center first due to breast cancer, and routine chest CT revealed lung nodules, which were pathologically confirmed as lung adenocarcinoma (24/31, 77.5%). Therefore, chest CT is indispensable for a thorough diagnosis of breast cancer. Lung cancer is the malignancy with the highest mortality (the world standard mortality is 28.09/100 000). After pathological diagnosis, staging work‐up is critically important and directly related to the treatment outcome. Among the work‐up examinations, PET‐CT has important value for lung cancer patients, particularly patients who are candidates for surgery. Compared with traditional examinations, PET‐CT detected at least 8% more occult metastases,^14^ which, in turn, led to avoidance of unnecessary and invalid surgery. Our primary lung cancer database clearly demonstrated that pretreatment PET‐CT detected almost all synchronous primary lung cancer and other primary solid tumors. In this study, we found that seven patients (7/31, 22.5%) who visited the thoracic surgery department first due to lung cancer were later diagnosed by PET‐CT to have synchronous breast cancer. Therefore, PET‐CT is indispensable for the thorough and accurate diagnosis of lung cancer.

### Interdisciplinary and multidisciplinary discussions are necessary to manage synchronous multiple primary cancers

Multidisciplinary management represents significant progress in oncology of solid tumors in the past 20 years, bringing favorable results in treating various tumors and continuously improving the treatment outcome.^15^, ^16^, ^17^ However, in a narrow sense, the multidisciplinary team is usually referred to different specialties engaged in the management of the same cancer type. For example, with lung cancer specialty, the multidisciplinary team includes lung cancer surgeons, oncologists, radiologists, and physicians who diagnose lung cancer. However, breast cancer and lung cancer belong to different branches of oncology regardless of diagnosis and treatment. Lung cancer surgery and breast cancer surgery belong to two distinct specialties. There is little overlap between the medical professionals of the two specialties, and there is a gap in knowledge categories. Since 1894 when humans began trying to treat breast cancer, treatment has made great progress, which is reflected in the continuous maturity of molecular classification; as well, the corresponding treatment strategies derived from molecular classification have constantly improved, including endocrine therapy for hormone receptor positive breast cancer, targeted treatment for patients with positive epidermal growth factor Her2, and chemotherapy for patients with triple‐negative breast cancer. Clearly, the status of systemic therapy in treating breast cancer has been greatly enhanced, and the status of surgery, especially the traumatic surgery represented by “extended resection”, has been declining. The treatment of lung cancer has gone through a similar development process since the first successful pneumonectomy in 1933. Currently (1) Stage 0 lung adenocarcinoma, which is manifested as pure GGO on thin‐layer high‐resolution CT (mostly lepidic growth under microscopic pathology), located in the lung parenchyma (isolated), and a diameter of <3 cm, is considered a special type of lung cancer with “indolent” growth, even several years without requiring immediate surgery.^7^ (2) For peripheral early stage solid lung cancer (stage IB or earlier) without enlarged hilar or bilateral mediastinal lymph nodes, the surgical procedures have also shifted from lobectomy to “smaller” procedures.^18^, ^19^ (3) For traditional early stage lung cancer with surgical indications (stage Ic–IIIa), principally, preoperative systematic treatment followed by surgery is emphasized.^10^, ^11^ (4) Unresectable and even stage IV lung cancer patients are recommended to receive efficacious systemic treatment followed by supplementary local treatment. The foregoing concepts are unfamiliar to breast cancer specialists and difficult for them to distinguish. Breast surgeons even adhere to the narrow thinking of “lung cancer is a highly lethal cancer”. When encountering lung cancer patients, breast surgeons often simply refer the patients to lung cancer specialists. Therefore, interdisciplinary discussion is necessary to confront synchronous primary breast cancer and primary lung cancer.

### Emphasize systemic treatment and arrange the treatment sequence

As mentioned earlier, when breast cancer and lung cancer occur in the same patient synchronously, inter‐ and multidisciplinary discussion should be considered.

First, in terms of mortality, generally, treating lung cancer should precede the treatment of breast cancer. However, if lung cancer is an “indolent” adenocarcinoma, the principle should be breast cancer treatment first and indolent lung cancer treatment second. Treating indolent lung cancer can be postponed for a long time, even several years. In this study, two patients had their lung cancer surgery more than 1 year after the completion of breast cancer treatment. If the breast and lung cancer were both indolent (both in very early stages) but needed surgery, the treatment can be performed simultaneously, even by one‐stage surgery.

Second, on the basis of the lung cancer treatment first and breast cancer treatment second principle, if preoperative therapy is needed for lung cancer, then the regimen should be balanced for breast cancer, including endocrine therapy, targeted therapy, and chemotherapy. Endocrine therapy for breast cancer does not increase the toxic side effects of any systemic therapy (chemotherapy or immunotherapy) for lung cancer. The side effects of targeted therapy for growth factor receptor combined with systemic therapy for lung cancer/breast cancer are also in a safe range. Lastly, chemotherapy for breast cancer and lung cancer can be balanced with mutual consideration. On the basis of lung cancer treatment first and breast cancer treatment second principle, whether surgery or systemic treatment followed by surgery for lung cancer could also take into consideration for the treatment of breast cancer, including endocrine therapy and targeted therapy. Although principally some breast cancer patients do not need endocrine therapy, the endocrine or targeted therapy during the treatment of lung cancer should be regarded as the preoperative treatment for lung cancer.

In summary, chest CT should be a routine examination for breast cancer patients, and PET‐CT is indispensable for the staging work‐up of lung cancer patients. The treatment strategy for synchronous primary lung cancer and primary breast cancer should be formulated after interdisciplinary team consultation. Priority should be given to patients who need systemic treatment followed by surgery, and preoperative treatment based on chemotherapy should balance the treatment for the two types of cancers.

## AUTHOR CONTRIBUTIONS

Xue Chen contributed the concept of the study and collected all the data for the database. Yi‐fan Fang performed the data analyses. Wan‐Pu Yan contributed to the trial design and data analyses.

## CONFLICT OF INTEREST STATEMENT

The authors declare no conflicts of interest.
